# A Human-Health Risk Assessment for West Nile Virus and Insecticides Used in Mosquito Management

**DOI:** 10.1289/ehp.8667

**Published:** 2005-10-28

**Authors:** Robert K.D. Peterson, Paula A. Macedo, Ryan S. Davis

**Affiliations:** Agricultural and Biological Risk Assessment, Department of Land Resources and Environmental Sciences, Montana State University, Bozeman, Montana, USA

**Keywords:** comparative risk assessment, mosquito control, organophosphates, pesticide exposure, pyrethroids, risk analysis, vectorborne disease

## Abstract

West Nile virus (WNV) has been a major public health concern in North America since 1999, when the first outbreak in the Western Hemisphere occurred in New York City. As a result of this ongoing disease outbreak, management of mosquitoes that vector WNV throughout the United States and Canada has necessitated using insecticides in areas where they traditionally have not been used or have been used less frequently. This has resulted in concerns by the public about the risks from insecticide use. The objective of this study was to use reasonable worst-case risk assessment methodologies to evaluate human-health risks for WNV and the insecticides most commonly used to control adult mosquitoes. We evaluated documented health effects from WNV infection and determined potential population risks based on reported frequencies. We determined potential acute (1-day) and subchronic (90-day) multiroute residential exposures from each insecticide for several human subgroups during a WNV disease outbreak scenario. We then compared potential insecticide exposures to toxicologic and regulatory effect levels. Risk quotients (RQs, the ratio of exposure to toxicologic effect) were < 1.0 for all subgroups. Acute RQs ranged from 0.0004 to 0.4726, and subchronic RQs ranged from 0.00014 to 0.2074. Results from our risk assessment and the current weight of scientific evidence indicate that human-health risks from residential exposure to mosquito insecticides are low and are not likely to exceed levels of concern. Further, our results indicate that, based on human-health criteria, the risks from WNV exceed the risks from exposure to mosquito insecticides.

West Nile virus (WNV) has become a major public health concern in North America since 1999, when the first outbreak in the Western Hemisphere occurred in New York City, causing 62 cases of human encephalitis and 7 deaths [Centers for Disease Control and Prevention (CDC) 1999]. The initial outbreak in New York City is thought to have affected 2.6% of the population (Hubalek 2001). In 2000, WNV spread to three states, with 21 human cases of WNV infection and 2 deaths. In 2001, 66 human cases and 9 deaths were reported in 10 states, before WNV spread westward, affecting all but 6 states in 2002 and causing the largest arboviral encephalitis epidemic in U.S. history (Huhn et al. 2003). A total of 4,156 human cases were documented, with 284 deaths reported (CDC 2003), and numbers continued to grow in 2003, when 46 states reported 9,862 human cases with 264 deaths (CDC 2004a). In 2004, 2,539 human cases and 100 deaths were reported in 41 states (Hayes et al. 2005). Since the first appearance of WNV in the United States in 1999, the CDC has reported 16,706 documented human cases and 666 deaths (CDC 2004b; Hayes et al. 2005); however, large numbers of human infections may not be detected because of significant underreporting of milder cases of West Nile fever (Hubalek 2001; Huhn et al. 2003). Given the infection rate observed for previous years, Peleman (2004) estimated that 1.5 million people were infected with the virus in 2003.

As a result of this ongoing disease outbreak, management of mosquitoes that vector WNV throughout the United States and Canada has necessitated using insecticides in areas where they traditionally have not been used or have been used less frequently. This practice has raised concerns by the public about risks from insecticide use. In a survey by Hinten (2000), 54% of 880 people surveyed were either equally afraid of WNV and pesticides or were more afraid of the insecticides. Since 1999, numerous concerns have been raised by the public regarding the safety of using insecticides to control mosquitoes (Cohen 2003; Fehr-Snyder 2004; Fitz 2003). Some of those concerned have even suggested that the health risks from the insecticides exceed those of WNV (Cohen 2003; Ziem 2005). These concerns by the public are not exclusive to the WNV issue, but reflect longstanding perceptions of risk from pesticides (Peterson and Higley 1993; Slovic 1987).

Risk assessment is a formalized basis for the objective evaluation of risk in which assumptions and uncertainties are clearly considered and presented [National Research Council (NRC) 1983, 1996]. Human-health and ecologic risk can be described in quantitative terms as a function of effect (also termed “hazard” or “toxicity”) and exposure (NRC 1983). Risk assessment typically uses a tiered modeling approach extending from deterministic models (tier 1) based on conservative assumptions to probabilistic models (tier 4) using refined assumptions [Society for Environmental Toxicology and Chemistry (SETAC) 1994]. In risk assessment, conservative assumptions in lower-tier assessments represent overestimates of effect and exposure; therefore, the resulting quantitative risk values typically are conservative and err on the side of safety.

Unfortunately, few, if any, science-based considerations of the risks of insecticide use versus the risks from vectorborne diseases have been examined. An understanding of the human-health risks for both vectorborne diseases and associated vector controls would aid greatly in decision making by all stakeholders. Therefore, the objective of this study was to use risk assessment methodologies to evaluate human-health risks from WNV and from the insecticides used to control adult mosquitoes.

## Materials and Methods

### Problem formulation.

Although WNV has important effects on horses and birds, our assessment of health risks from WNV focused only on humans. Currently, effect and exposure factors for WNV are poorly understood (Loeb et al. 2005), making quantitative modeling of risk difficult. Therefore, we evaluated documented health effects from WNV infection and determined potential population risks based on reported frequencies. Because of the relatively recent emergence of WNV in North America, information on prevalence of various effects of the disease should be regarded as tentative.

Our tier-1 quantitative assessment of human-health risks associated with insecticides used in mosquito control focused on acute and subchronic residential exposures after truck-mounted ultra-low-volume (ULV) spraying of mosquito adulticides. The dissemination of mosquito adulticides by ULV application generates fine aerosol droplets that remain aloft and target flying mosquitoes [U.S. Environmental Protection Agency (EPA) 2002b]. Acute exposures were defined as single-day exposures immediately after a spray event. Subchronic exposures were defined as exposures per day over a 90-day seasonal multispray event. A total of 10 spray events were assumed to occur on days 1, 4, 14, 17, 27, 30, 40, 43, 53, and 56. This design represents a reasonable worst-case mosquito insecticide seasonal application scenario, including during a human epidemic of WNV [Karpati et al. 2004; New York City Department of Health (NYCDOH) 2001]. Chronic exposures (> 6 months) to mosquito adulticides are unlikely. Additionally, extrapolation of subchronic exposures to chronic exposure time frames would result in lower risks than with subchronic risks (NYCDOH 2001). Therefore, chronic risks were not assessed in this study.

Exposures to several population subgroups were estimated to account for potential age-related differences in exposure. Groups included adult males, adult females, infants (0.5–1.5 years of age), and children (2–3, 5–6, and 10–12 years of age). Adult males were assumed to weigh 71.8 kg, which represents the mean body weight for all males > 18 years of age, and adult reproductive females were assumed to weigh 60 kg, which represents the mean body weight for females between 13 and 54 years of age (U.S. EPA 1996). Children 5–6 and 10–12 years of age were assumed to weigh 21.1 and 40.9 kg, respectively. Infants (0.5–1.5 years of age) and toddlers (2–3 years of age) were assumed to weigh 9.4 and 14.3 kg, respectively. All weights for children were derived from mean body weight values for male and female children within their respective age groups (U.S. EPA 1996).

### Hazard identification.

We conducted human-health risk assessments for six insecticide active ingredients (permethrin, pyrethrins, resmethrin, phenothrin, malathion, and naled) and one synergist (piperonyl butoxide). Malathion and naled are in the organophosphate class of insecticides, and permethrin, pyrethrins, resmethrin, and phenothrin are in the pyrethroid class. The synergist, piperonyl butoxide, is present in many formulations with pyrethroids. All compounds are currently registered by the U.S. EPA for adult mosquito management in the United States.

#### Toxicity end points.

Toxicity and dose–response information for each compound were reviewed for acute and subchronic exposure durations. Toxicity end points in this assessment were chosen based on U.S. EPA regulatory end points. We used inhalation, dermal, and ingestion toxicity end points for each respective exposure route and duration. Ingestion reference doses (RfDs) were used as the toxicity end points (acceptable daily exposures) and were compared with total estimated exposures (total body burden). Acute and subchronic ingestion RfDs were calculated by dividing the most sensitive toxic effect [typically the no observed adverse effect level (NOAEL)] by a series of uncertainty factors (typically a factor of 100 to account for intraspecies and inter-species uncertainty) (Table 1).

### Environmental concentrations and fate of insecticides.

We used the AERMOD, version 1.0 tier 1 air dispersion model (U.S. EPA 1999) to predict the 7.6 m (25 ft) and 91.4 m (300 ft) air concentrations (micrograms per cubic meter) of each insecticide within 1- and 6-hr time ranges after ULV application by a truck-mounted sprayer. Estimates of environmental concentrations are presented only for truck-mounted ULV applications because our modeling suggested that delivery of ULV applications by aircraft resulted in substantially less aerial and surface deposition (and therefore less human exposure and risk). This was also observed by the NYCDOH (2001).

We used the following conservative assumptions: *a*) each chemical had a 24-hr half-life in air except for naled, which was given a 18-hr half-life; *b*) the insecticides were applied at the maximum application rate as stated on each label; *c*) all of the insecticides were susceptible to the same weather conditions using standardized weather data from Albany, New York, in 1988; *d*) all spray events occurred at 2100 hr; and *e*) each spray release was at 1.5 m. The chemical properties, application rates, and predicted environmental concentrations for each active ingredient are listed in Table 2.

Receptors were established within the model on a Cartesian grid at five intervals of 25 m at 7.6 m and 91.4 m from the edge of the spray emission area. The receptors were at a height of 1.5 m. Each receptor estimated the 1-and 6-hr average air concentrations for each insecticide. An average was then taken of the estimates from the six receptors at 7.6 m that were not at the edges of the spray zone. The following data were obtained using this network of receptors: the 1-hr average concentration at 7.6 m, the 6-hr average at 7.6 m, and the peak value at 91.4 m.

We used the screening Industrial Source Complex Short-Term (ISCST3) model (U.S. EPA 1995) to estimate particle deposition (milligrams per square meter) at 7.6 m and 91.4 m from the spray area at a 1-hr average. The following assumptions were made in addition to those from AERMOD: *a*) all of the insecticides were susceptible to the same weather conditions using standardized weather data from Salem, Massachusetts; *b*) the ULV particle size applications had 3% of the emitted particles greater than the allowable particle size as stated on the label; and *c*) the particles were assigned a density in accordance with the specific gravity of each insecticide.

A Cartesian grid was used for ISCST3 that was similar to that used in AERMOD. Receptors were added at 15.24-m intervals between 7.6 m and 91.4 m from the spray source to obtain a more accurate estimate of the average deposition within 91.4 m of the source. The receptors were also at the same height of 1.5 m. All of the same methods were used to calculate the average deposition at 7.6 m and 91.4 m. The middle receptors were included to calculate an average deposition within 91.4 m. The following data were obtained from this information: deposition at 7.6 m, deposition at 91.4 m, and the average deposition within 91.4 m of the spray source.

For estimating subchronic exposures, we used the estimated deposition values within 91.4 m for each insecticide in an exponential decay model to characterize their persistence on surfaces such as soil within a spray program that included 10 sprays on days 1, 4, 14, 17, 27, 30, 40, 43, 53, and 56. Insecticide concentrations for each spray event were followed through day 90 using the following multiple degradation model:


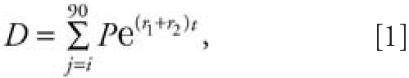


where *D* is the sum of the deposition over one spray, *P* is the peak deposition after a spray event, *r_1_* is the rate of decay calculated by using the aerobic soil half-life of each active ingredient, *r_2_* is the rate of decay calculated by using the soil photolysis half-life of each active ingredient, *t* is the time in hours, and *j* is the spray day. The average daily exposure was then determined by dividing the deposition sum by 90.

The same deposition and degradation model was used to characterize deposition and persistence on garden produce by using a Kenaga nomogram to estimate the deposition (milligrams per kilogram dry weight) of each insecticide on respective plant parts. Because the nomogram represents a linear relationship between application rate and maximum residues, it can be used to estimate the maximum residues on plant surfaces for a given application rate (Fletcher et al. 1994). For this analysis, maximum application rates were used for each insecticide, and each estimated concentration was then applied to the model above, using the surface photolysis half-life to estimate the rate of degradation.

### Acute exposure.

We assumed that multi-route exposures immediately after a single-spray event were limited to 24 hr. Routes of insecticide exposure included inhalation, dermal contact with spray, hand-to-mouth ingestion by infants and toddlers from spray deposition on hands, and ingestion of garden produce. We also assumed that residents did nothing to limit their exposure to the spray. In its assessment of acute and subchronic exposures from several mosquito adulticides, the NYCDOH (2001) concluded that exposures from potable water and swimming were negligible. Using environmental fate models, we also concluded that the chemical properties of the insecticides result in negligible concentrations in water. Therefore, we did not include these exposures in our assessment.

#### Acute inhalation exposure.

Acute inhalation exposures were estimated as


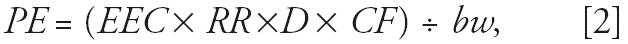


where *PE* is potential exposure [milligrams per kilogram body weight (bw)], *EEC* is the 6-hr average estimated environmental concentration of an active ingredient in the air 1.5 m high at 7.6 m from the spray source (micrograms per cubic meter), *RR* is the respiratory rate under moderate activity (cubic meters per hour), *D* is the duration of exposure (hours), *CF* is the conversion factor to account for the conversion of units from micrograms per cubic meter to milligrams per cubic meter, and *bw* is body weight (kilograms).

RRs were assumed to be 1.6 m^3^/hr for adults and 1.2 m^3^/hr for children, including infants. These rates are indicative of moderate physical activity (U.S. EPA 1996). The duration of exposure was 6 hr. Therefore, the assumption was that the person was outside and 7.6 m from the spray truck when it passed him or her. Moreover, the person remained outside, 7.6 m from the emission, for the following 6 hr, respiring as if under moderate physical activity during the entire time. Body weight for the different age groups is discussed above.

#### Acute dermal exposure from spray deposition.

Acute dermal exposures from deposition of spray drift on skin were estimated as


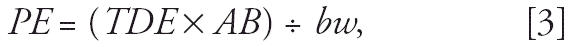


where *PE* is potential exposure (milligrams per kilogram bw), *TDE* is total dermal exposure (milligrams), *AB* is dermal absorption rate, and *bw* is body weight (kilograms). There is no publicly available information on dermal deposition immediately after truck-mounted ULV sprays. Therefore, we used the U.S. EPA Pesticide Handler Exposure Database (PHED; U.S. EPA 1998) as a conservative surrogate. The PHED contains pesticide-handler scenarios derived from field studies and exposure estimates based on physical factors such as application rate, hectares treated per day, type of clothing worn, methods of application, and formulation type. We used the PHED scenario in which a flagger (person marking the location for pesticide application while the application is occurring) was exposed to a liquid application. We assumed that the person was not wearing clothing and that the exposure was 10 times greater than the flagger scenario. We believe this scenario conservatively estimated residential dermal exposure for two reasons: *a*) we added a 10-fold increase in exposure, and *b*) the U.S. EPA has not considered acute dermal contact from ULV applications for pyrethrins, piperonyl butoxide, and permethrin because it was believed to be negligible (U.S. EPA 2005a, 2005b, 2005c). The values for percent dermal absorption were 0.22% for pyrethrins (U.S. EPA 2005b), 2% for piperonyl butoxide (U.S. EPA 2005a), 10% for malathion and resmethrin (U.S. EPA 2000a, 2000c), 15% for permethrin (U.S. EPA 2005c), 70% for phenothrin (U.S. EPA 2000b), and 100% for naled (U.S. EPA 2002a).

#### Acute hand-to-mouth exposure from spray deposition on hands.

Acute hand-to-mouth exposures were estimated for only two subgroups (toddlers and infants), because young children are more likely than adults to be exposed to pesticides as a result of hand-to-mouth contact (Cohen Hubal et al. 2000). Exposures were calculated as


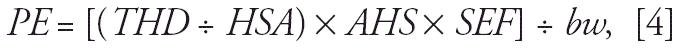


where *PE* is potential exposure (milligrams per kilogram bw), *THD* is total hand dermal exposure (milligrams), *HSA* is adult hand surface area (square meters), *AHS* is adjusted hand surface area for each subgroup (square meters), *SEF* is saliva extraction factor, and *bw* is body weight (kilograms). Total hand dermal exposure was determined using the PHED database and the assumptions discussed above. The hand surface area of toddlers (2–3 years of age) was assumed to be 0.035 m^2^, which represents the 50th percentile total surface area values for males and females in the 2–3 and 3–4 year age groups, multiplied by the mean percentage of the total body represented by hands for males and females of that age (U.S. EPA 1996). The hand surface area for infants was assumed to be 0.007 m^2^ and was also calculated as a percent of total body surface area for infants (U.S. EPA 1996). We calculated the total body surface area of infants using the formula by Current (1998). We assumed that, on the day of application, 50% of the insecticide deposited on the hand was available through saliva extraction (U.S. EPA 2005a, 2005c).

#### Acute ingestion of garden produce.

We assumed that the insecticide settled onto a tomato garden and that the resident picked, processed, and ate tomatoes the next day. The estimated maximum insecticide residue deposited on tomatoes is discussed above. We assumed that the resident did not wash the tomatoes after picking. The residue concentration also did not change with processing of the tomatoes. The amount of insecticide ingested was estimated as the product of the residue concentration and the quantity of food consumed. Tomato consumption patterns were determined using the Dietary Exposure Evaluation Model [(DEEM)-Food Commodity Intake Database (FCID) version 2.04; Exponent, Washington, DC]. The model determines dietary consumption for the U.S. population and several subgroups by using individual food consumption records collected by the U.S. Department of Agriculture (USDA) Continuing Surveys for Food Intake by Individuals for 1994–1998. Translation factors used to convert foods-as-eaten to commodities are based on a U.S. EPA/USDA FCID recipe set. For this assessment, we determined the acute food consumption patterns by subgroup using the 95th percentile 1-day consumption values for tomatoes, tomato baby food, tomato paste, tomato paste baby food, tomato puree, tomato puree baby food, dried tomato, dried tomato baby food, and tomato juice. Therefore, the respective individuals in these subgroups ate all of these tomato food products within 1 day of application at the 95th percentile of U.S. national consumption.

### Subchronic exposure.

We assumed multi-route exposures per day over 90 days after multispray events. Routes of insecticide exposure included inhalation, dermal contact with spray, ingestion of garden produce, hand-to-mouth ingestion by infants and toddlers from spray deposition on hands, hand-to-mouth ingestion by infants and toddlers from deposition on surfaces, dermal contact with soil and other surfaces, and soil ingestion.

#### Subchronic inhalation, dermal, and hand-to-mouth exposures.

Exposures for each exposure type were estimated as


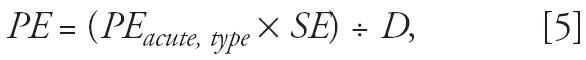


where *PE* is the potential exposure (milligrams per kilogram bw per day), *PE_acute, type_* is the acute exposure type (e.g., acute inhalation) from each spray event (milligrams per kilogram bw), *SE* is the number of spray events, and *D* is the duration of exposure (days). We assumed that the insecticides were sprayed on days 1, 4, 14, 17, 27, 30, 40, 43, 53, and 56 (10 spray events per season) in any given area. The exposure duration was 90 days.

#### Subchronic hand-to-mouth exposure from deposition on surfaces.

Subchronic hand-to-mouth exposures were estimated for only two subgroups (toddlers and infants) based on the rationale discussed above. Exposures were calculated as


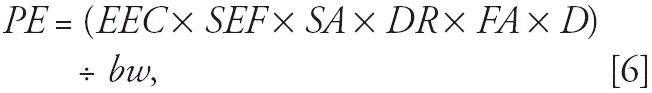


where *PE* is potential exposure (milligrams per kilogram bw per day), *EEC* is the 90-day average environmental concentration of the active ingredient deposited on soil or turf within 91.4 m from the spray source (milligrams per square meter), *SEF* is saliva extraction factor, *SA* is surface area for three fingers (square meters), *DR* is dislodgeable residue, *FA* is frequency of activity (events per hour), *D* is exposure duration (hours), and *bw* is body weight (kilograms). Assumptions for estimating subchronic environmental concentrations are discussed above. The saliva extraction factor was assumed to be 50% (U.S. EPA 2005a, 2005c), and the palmar surface area for three fingers was assumed to be 20 cm^2^ (U.S. EPA 2005c). Dislodgeable insecticide residue from soil or turf grass was assumed to be 20% (U.S. EPA 1997). The frequency of hand-to-mouth activity in children was assumed to be 20.5 events/hr and is based on the maximum frequency observed (Freeman et al. 2005). The duration of exposure was assumed to be 4 hr/day. Therefore, the toddler or infant was assumed to be engaging in hand-to-mouth activities outside each day for 4 hr over 90 days.

#### Subchronic ingestion of garden produce.

Our assumptions for subchronic ingestion of garden produce were the same as for acute ingestion of produce, with the following differences: *a*) the insecticide was deposited onto both tomatoes and head- and leaf-lettuce, *b*) all tomato and lettuce consumption by the residents over the 90 days was from the garden, and *c*) tomato and lettuce consumption patterns were determined using chronic food consumption patterns (3-day average).

#### Subchronic dermal contact with soil and other surfaces.

Exposures from contact with soil, turf, and other outdoor surfaces were calculated as


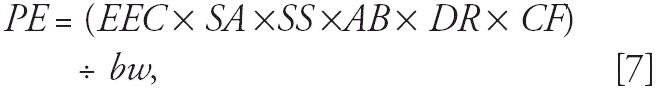


where *PE* is potential exposure (milligrams per kilogram bw per day), *EEC* is the 90-day average environmental concentration of the active ingredient deposited on soil or turf within 91.4 m from the spray source (milligrams per square meter), *SA* is body surface area in contact with surface (square centimeters), *SS* is weight of soil adhered to skin (milligrams per square centimeter), *AB* is dermal absorption rate, *DR* is dislodgeable residue, *CF* is the conversion factor to account for square meters to square centimeters, and *bw* is body weight (kilograms). The body surface area in contact with the surface was assumed to be the sum of surface areas for face (head ÷ 2), hands, arms, legs, and feet (U.S. EPA 1996). Therefore, we assumed residents were minimally clothed while outside. Contact with surfaces was associated with certain human activities. The activities were assumed to be gardening for adults (0.55 mg soil/cm^2^ skin) and soccer for children, including infants (0.164 mg soil/cm^2^ skin) (U.S. EPA 1996). We assumed that these activities occurred each day over the 90 days. The assumptions for dermal absorption rate and dislodgeable residues are discussed above.

#### Subchronic soil ingestion.

Exposures from incidental ingestion of soil were calculated as


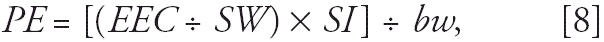


where *PE* is potential exposure (milligrams per kilogram bw per day), *EEC* is the 90-day average environmental concentration of the active ingredient deposited on soil or turf within 91.4 m from the spray source (milligrams per square meter), *SW* is soil weight (milligrams per cubic meter), *SI* is soil ingestion (milligrams per day), and *bw* is body weight (kilograms). Because the insecticide would only be surface-deposited on soil, we assumed that the concentration (milligrams per square meter) would be the same for a cubic meter of soil. Soil weight was assumed to be 3.86 kg/m^3^ based on reported densities for Scotts lawn soil (The Scotts Company, Marysville, OH). Soil ingestion rates were assumed to be 100 mg/day for children and 50 mg/day for adults (U.S. EPA 1996). We assumed that all soil ingestion each day was from soil containing residues of the active ingredients.

### Risk characterization.

Human-health risks in this study were assessed by integrating toxicity and exposure. We assessed risks using the risk quotient (RQ) method. For each population subgroup, an RQ was calculated by dividing the PE by the appropriate toxicity end point (e.g., the RfD). Therefore, the RQ is the ratio of exposure to effect. RQs < 1 are typically below regulatory levels of concern.

Exposures by similar route of exposure and duration (e.g., subchronic dermal contact with spray and surfaces) were compared with the appropriate RfD (e.g., subchronic dermal RfD). Multiroute exposures (dermal + ingestion + inhalation) were compared with the ingestion RfD. The ingestion RfD provided a conservative toxicity end point because it typically was based on the most sensitive NOAEL. Therefore, it represented the largest dose in which no adverse effects on human health would occur during the relevant exposure duration.

## Results

### West Nile virus risks.

According to a sero-epidemiologic survey conducted by Mostashari et al. (2001), for every diagnosed case of West Nile (WN) meningoencephalitis, there were approximately 30 additional people with WN fever, and approximately 2.6% of the population in outbreak areas in New York were infected during the epidemic of 1999. Loeb et al. (2005) reported a 3.1% outbreak infection rate in Oakville, Ontario, Canada, in 2002. Unfortunately, the seroprevalence of WNV antibodies across larger time and geographic scales has not been determined. Overall, 20% of infected persons develop mild febrile illness (Mostashari et al. 2001), and 0.67% develop neurologic disease (Fratkin et al. 2004). A total of 0.43% develop encephalitis, and 0.24% develop meningitis (Asnis et al. 2001; Brilla et al. 2004; Emig and Apple 2003; Klee et al. 2004; Sejvar et al. 2003a; Weiss et al. 2001).

Case-fatality rates in the United States ranged from 4 to 18% among hospitalized patients (Brilla et al. 2004; Emig and Apple 2003; Nash et al. 2001b; Pepperell et al. 2003; Sejvar et al 2003a; Weiss et al. 2001) and from 2.7 to 14% among cases reported to the CDC (CDC 2004b).

No difference in distribution of WNV infection among age groups and between sexes is apparent (Nash et al. 2001a, 2001b; Tyler 2001), but for unknown reasons, males seem to be at higher risk for WN neuroinvasive illness (O’Leary et al. 2004; Petersen and Marfin 2002). Children infected with WNV usually show no symptoms or have only a mild fever (Hayes and O’Leary 2004). The incidence of encephalitis and death increases with age (Nash et al. 2001a, 2001b; O’Leary et al. 2004; Tsai et al. 1998; Weinberger et al. 2001). Weiss et al. (2001) reported that persons ≥ 50 years of age were more likely to present meningo-encephalitis and had increased mortality rates; other reports show that the incidence of neurologic symptoms and death may increase 10- to 20-fold among persons ≥ 50 years of age (Nash et al. 2001a; Sampathkumar 2003; Tyler 2001). The risk increases 43 times for persons ≥ 80 years of age (Sampathkumar 2003).

Few data exist regarding long-term morbidity after WNV infection. Substantial morbidity may follow hospitalization for WNV infection (Petersen et al. 2003) and is observed in patients with WN fever (Watson et al. 2004). Encephalitis cases seem to have more variable outcomes than meningitis cases, which tend to recover well (Granwehr et al. 2004). A poor prognosis and very limited recovery have been observed in acute flaccid paralysis cases (Saad et al. 2005; Sejvar et al. 2003a, 2003b).

Although patients with WN fever tend to recover well, median recovery time was 60 days for patients in Illinois in 2002 (Watson et al. 2004). The disease also has a significant effect on the lifestyle of patients with WN fever. Of 98 respondents with WN fever, 57 (58%) missed work/school, 82 (84%) had household activities limited, 47 (49%) had difficulty walking, and 89 (91%) had outside-of-home activities limited (Watson et al. 2004).

In a long-term follow-up study on 42 WN encephalitis survivors 1 year after illness onset, only 37% presented full physical, functional, and cognitive recoveries, and there was a substantially higher prevalence of impairment compared with baseline (Nash et al. 2001a). Similarly, only 2 of 8 patients in a study in New York presented full recovery after 1 year; 3 patients had neurologic sequelae, and 1 patient had minimal impairment after 18 months (Asnis et al. 2001).

### Acute risks from insecticides.

Table 3 shows the calculated RQs for each active ingredient in terms of total acute PE. Exposures and risks also were determined for each exposure route. Potential acute inhalation exposures of the six human subgroups to the adulticides ranged from 0.00011 to 0.0075 mg/kg bw, and the environmental concentrations were lower than the inhalation reference concentrations for all active ingredients evaluated. Potential acute dermal exposures to the adulticides ranged from 0.0000001 to 0.0011 mg/kg bw, with RQs ranging from 0.0000005 to 0.0113. For acute exposure due to ingestion (hand-to-mouth exposure from spray deposition on hands and ingestion of produce), total PEs ranged from 0.0001 to 0.0061 mg/kg bw, with RQs ranging from 0.00014 to 0.2142. Total acute RQs ranged from 0.0004 to 0.4726.

### Subchronic risks from insecticides.

Table 4 shows the calculated RQs for each active ingredient in terms of total subchronic PE. Potential subchronic inhalation exposures of the six subgroups to the adulticides ranged from 0.000012 to 0.00083 mg/kg bw. For subchronic dermal exposures to the adulticides (dermal and contact with soil), total PEs ranged from 0.00000006 to 0.00015 mg/kg, with RQs ranging from 0.0000001 to 0.0015. Potential subchronic exposures due to ingestion (ingestion of produce and soil, hand-to-mouth activity after contact with surfaces, and hand-to-mouth activity after contact with spray drift) ranged from 0.00001 to 0.0283 mg/kg bw, with RQs ranging from 0.00007 to 0.1709. Total subchronic RQs ranged from 0.00014 to 0.2074.

None of the subgroups had RQs ≥ 1.0 (i.e., PEs did not equal or exceed the RfDs) for any of the active ingredients evaluated. The lowest acute RQs were to phenothrin and piperonyl butoxide for adults and the highest acute RQ was to naled for toddlers (Table 3). The lowest and highest subchronic RQs were to phenothrin for adults and malathion for infants, respectively (Table 4).

## Discussion

### Conservatism.

Based on the exposure and toxicity assumptions above, we believe our assumptions were sufficiently conservative and most likely overestimated risk. For example, assuming an acute RR of 0.8 m^3^/hr for 2 hr and no dermal or ingestion exposures [which were the U.S. EPA assumptions for mosquito control uses of permethrin (U.S. EPA 2005c)], there would be a 90% reduction in exposure for toddlers compared with our value. Indeed, draft tier 1 risk assessments recently conducted for malathion, piperonyl butoxide, pyrethrins, and permethrin by the U.S. EPA also suggest that our results are sufficiently conservative (U.S. EPA 2000c, 2005a, 2005b, 2005c). Because of the conservative exposure assumptions used, we believe higher-tiered risk assessments using more realistic exposures would result in risk values significantly lower than those presented here.

The conservatism of our risk assessments for insecticides used in adult mosquito control is supported by residential biomonitoring and epidemiologic studies. Currier et al. (2005) assessed human exposure to ULV-applied naled, permethrin, and phenothrin in Mississippi, North Carolina, and Virginia as a result of emergency large-scale mosquito abatement. Using biomonitoring of urine, they did not observe an increase in insecticide metabolite concentrations among exposed residents. Karpati et al. (2004) and O’Sullivan et al. (2005) did not observe increases in hospital emergency department visits for asthma after wide-scale spraying of residential neighborhoods.

### Uncertainties.

Despite the conservatism of our risk assessment, uncertainties were revealed. Many of the uncertainties associated with residential exposure estimates are discussed above. The principal uncertainty was for environmental concentrations of the active ingredients. Data for actual aerial concentrations and surface deposition of active ingredients need to be generated to more accurately characterize risks. Because of the nature of ULV application methods, it is likely that concentrations of active ingredients are much lower than those predicted using the AER-MOD and ISCST3 tier 1 models. Toxicologic uncertainties include mammalian toxicities to combinations of piperonyl butoxide and adulticides and to inert ingredients in the formulated products. The addition of piperonyl butoxide to the adulticides increases the mosquito toxicity of the pyrethroids approximately 10-fold, but mammalian toxicity is not likely to be proportionally increased (Knowles 1991). Even if mammalian toxicity were increased 10-fold to the pyrethroids, RQs would still be well below levels of concern. Human exposures to solvents and other inert ingredients are likely to be low, resulting in low risks (NYCDOH 2001). Future research should be directed toward reducing toxicity and exposure uncertainties associated with mosquito adulticides. In addition, future assessments should address ecologic risks.

### Comparing risks.

Although it is difficult to compare the risks directly, several conclusions can be drawn by considering both human risks from exposure to WNV and insecticides used to control adult mosquitoes. In a situation where application of mosquito adulticides occurs because of known human cases of WNV, an adult human female may have at least a 3% probability of being infected by WNV. An adult female in that same area conservatively may have a 100% probability of being exposed to a particular mosquito adulticide. Her probability of exposure to the insecticide may be greater than WNV infection, but the consequences (i.e., the risks) of the exposures would be very different. Once infected with WNV, an adult human female has approximately a 20% probability of expressing clinical signs of illness (WN fever) and, depending on age, a 0.67% probability of expressing neurologic disease. Depending on the insecticide, her acute exposure would be 0.0415–15.76% of the RfD (0.0004–0.1576% of the NOAEL). Consequently, her acute risks from the insecticide would be lower than her acute risks from WNV. Subchronic insecticide risks would also be negligible (Table 4), whereas subchronic and chronic WNV risks (disease sequelae) would be greater. Therefore, once exposed to the insecticide (based on the tier 1 exposure assumptions from this study), the risk of any adverse health effects to the adult female would be negligible.

Results from our risk assessment and the current weight of scientific evidence (Currier et al. 2005; Karpati et al. 2004; NYCDOH 2001; O’Sullivan et al. 2005; U.S. EPA 2000c, 2005a, 2005b, 2005c) indicate that human-health risks from residential exposure to mosquito adulticides are very low and are not likely to exceed levels of concern. Further, by virtually any current human-health measure, the risks from infection by WNV exceed the risks from exposure to mosquito insecticides. Therefore, perceptions that human-health risks from the insecticides used to control adult mosquitoes are greater than the risks from WNV currently cannot be supported by current scientific evidence. Our results, and the results from other studies, should be used by the U.S. EPA, public health officials, and the general public to make better-informed decisions about risk–risk tradeoffs.

## Correction

In the original manuscript published online, the acute air concentration for naled in Table 2 and the RQ ranges for acute inhalation exposures and acute subchronic dermal exposures were incorrect. These have been corrected here.

## Figures and Tables

**Table 1:** Toxicologic effects and regulatory end points for the active ingredients.

	Acute		Subchronic	
Compound	End point	Study and toxicologic effects	End point	Study and toxicologic effects
Malathion	NOAEL = 50 mg/kg/day^a^ RfD = 0.5 mg/kg/day UF = 100	Based on reduction in maternal bw gain in a study with pregnant rabbits^a^	NOAEL = 2.4 mg/kg/day^a^ RfD = 0.024 mg/kg/day UF = 100	Based on inhibition of blood enzyme activity at 50 ppm malathion in the diet in a 24-month study in rats^a^
Naled	NOAEL = 1.0 mg/kg/day^b^ RfD = 0.01 mg/kg/day UF = 100	Based on inhibition of blood and brain enzymes in a 28-day study in rats^b^	NOAEL = 1.0 mg/kg/day^b^ RfD = 0.01 mg/kg/day UF = 100	Based on inhibition of blood and brain enzymes in a 28-day study in rats^b^
Permethrin	NOAEL = 25 mg/kg/day^c^ RfD = 0.25 mg/kg/day UF = 100	Acute neurotoxicity study in rats LOEL = 75 mg/kg based on observations of clinical signs such as aggression, abnormal/decreased movement, and increased body temperature^c^	NOAEL = 25 mg/kg/day^c^ RfD = 0.25 mg/kg/day UF = 100	Acute neurotoxicity study in rats LOEL = 75 mg/kg based on observations of clinical signs such as aggression, abnormal/decreased movement, and increased body temperature^c^
Resmethrin	NOEL = 10 mg/kg/day^d^ RfD = 0.1 mg/kg/day UF = 100	Based on liver weight increases in a 6-month study in dogs^d^	NOEL = 10 mg/kg/day^d^ RfD = 0.1 mg/kg/day UF = 100	Based on liver weight increases in a 6-month study in dogs^d^
Phenothrin	NOEL = 70 mg/kg/day^e^ RfD = 0.7 mg/kg/day UF = 100	13-week study in rats LOEL = 216 mg/kg-day based on increases in liver weights and decreases in cholesterol in both male and female rats^e^	NOEL = 70 mg/kg/day^e^ RfD = 0.7 mg/kg/day UF = 100	13-week study in rats LOEL = 216 mg/kg-day based on increases in liver weights and decreases in cholesterol in both male and female rats^e^
Pyrethrins	NOAEL = 20 mg/kg/day^f^ RfD = 0.07 mg/kg/day UF = 300	Acute neurotoxicity study in rats LOAEL = 63 mg/kg/day based on tremors in females^f^	NOAEL = 4.37 mg/kg/day^f^ RfD = 0.044 mg/kg/day UF = 100	Rat chronic toxicity study LOAEL = 42.9 mg/kg/day based on increased incidence of thyroid follicular cell hyperplasia in males.^f^
Piperonyl butoxide	NOAEL = 630 mg/kg/day^g^ RfD = 6.3 mg/kg/day UF = 100	Developmental toxicity study in rats LOAEL = 1,065 mg/kg/day based on decreases in maternal bw gain^g^	NOAEL = 89 mg/kg/day^g^ RfD = 0.89 mg/kg/day UF = 100	Two generation reproduction study in rats LOAEL = 469 mg/kg/day based on decrease in bw gain of F_1_ and F_2_ pups at postnatal day 2^g^

Abbreviations: bw, body weight; LOAEL, lowest observed adverse effect level. LOEL, lowest observed effect level; NOEL, no observed effect level; UF, uncertainty factor used to determine the RfD.

a U.S. EPA 2000c.

b U.S. EPA 2002a.

c U.S. EPA 2005c.

d U.S. EPA 2000a.

e U.S. EPA 2000b.

f U.S. EPA 2005b.

g U.S. EPA 2005a.

**Table 2:** Application rates, chemical properties, and predicted environmental concentrations of active ingredients.

	Active ingredient						
Property	Piperonyl butoxide	Phenothrin	Permethrin	Resmethrin	Malathion	Naled	Pyrethrins
Application rate (kg ai/ha)	0.0392	0.004	0.0078	0.0078	0.0639	0.0224	0.009
Density (g/mL)	0.898^a^	0.898^a^	0.8657^b^	0.87^c^	1.23^d^	1.67^e^	0.81^f^
Surface photolysis half-life (days)	NA^g^	6^c^	23^h^	0.14^i^	6.5^i^	2.4^i^	0.5^j^
Soil aerobic half-life (days)	14^i^	7^i^	37^k^	30^h^	1^h^	1^h^	1^j^
Acute air concentration (μg/m^3^)^l^	7.39	0.81	1.55	1.61	9.76	1.68	1.7
1-Day acute produce concentration (mg/kg dry wt)	0.525	0.054	0.105	0.105	0.855	0.3	0.12
90-Day mean surface concentration (mg/m^2^)^m^	15.42	0.43	4.14	0.22	2.18	0.65	0.54
90-Day mean produce concentration (mg/kg dry wt)	2.88	0.055	0.096	0.012	0.73	0.13	0.21

Abbreviations: ai/ha, active ingredient per hectare; NA, not available; wt, weight.

a Clarke Mosquito Control Products (1999b).

b Clarke Mosquito Control Products (1999a).

c Bayer Environmental Science (2004).

d Griffin (2001).

e AMVAC (2003).

f McLaughlin Gormley King Co. (2004).

g Surface and produce concentrations determined from soil aerobic half-life only.

h U.S. Department of Agriculture (USDA 2005).

i NYCDOH (2001).

j Food and Agricultural Organization (2000).

k U.S. EPA (2005c).

l 6-Hr mean concentration at 7.6 m from spray source.

m 90-Day mean surface concentration within 91.4 m of the spray source.

**Table 3:** Acute RQs for the active ingredients for each subgroup.^a^

Subgroup	Malathion	Naled	Permethrin	Resmethrin	Phenothrin	Pyrethrins	Piperonyl butoxide
Adult males	0.0076	0.1496	0.0020	0.0052	0.0004	0.0081	0.0004
Adult females	0.0079	0.1576	0.0021	0.0055	0.0004	0.0085	0.0004
Children (10–12 years)	0.0105	0.2123	0.0029	0.0072	0.0006	0.0113	0.0006
Children (5–6 years)	0.0177	0.3631	0.0049	0.0123	0.0010	0.0190	0.0009
Toddlers (2–3 years)	0.0225	0.4726	0.0063	0.0159	0.0013	0.0245	0.0012
Infants (0.5–1.5 years)	0.0188	0.4495	0.0058	0.0147	0.0012	0.0218	0.0010

a RQ = total acute PE ÷ RfD.

**Table 4:** Subchronic RQs for the adulticides for each subgroup.^a^

Subgroup	Malathion	Naled	Permethrin	Resmethrin	Phenothrin	Pyrethrins	Piperonyl butoxide
Adult males	0.0360	0.0259	0.0007	0.0004	0.0001	0.0056	0.0032
Adult females	0.0363	0.0269	0.0007	0.0004	0.0001	0.0056	0.0032
Children (10–12 years)	0.0470	0.0290	0.0008	0.0005	0.0001	0.0074	0.0043
Children (5–6 years)	0.0676	0.0447	0.0012	0.0009	0.0002	0.0104	0.0059
Toddlers (2–3 years)	0.1815	0.1294	0.0204	0.0037	0.0009	0.0270	0.0262
Infants (0.5–1.5 years)	0.2074	0.1661	0.0301	0.0054	0.0013	0.0292	0.0325

a RQ = total subchronic PE ÷ RfD.
